# The Ayurvedic Medicine *Salacia oblonga* Attenuates Diabetic Renal Fibrosis in Rats: Suppression of Angiotensin II/AT1 Signaling

**DOI:** 10.1093/ecam/nep095

**Published:** 2011-06-08

**Authors:** Lan He, Yanfei Qi, Xianglu Rong, Jianmin Jiang, Qinglin Yang, Johji Yamahara, Michael Murray, Yuhao Li

**Affiliations:** ^1^School of Pharmaceutical Sciences, Sun Yat-Sen University, Guangzhou, 510006, China; ^2^Faculty of Pharmacy, The University of Sydney, Sydney, NSW 2006, Australia; ^3^Department of Pharmacology, Guangzhou University of Chinese medicine, Guangzhou, 510006, China; ^4^Department of Nutrition Sciences, University of Alabama, Birminham, 35294-3360, USA; ^5^Pharmafood Institute, Kyoto, 602-8136, Japan

## Abstract

In human diabetic nephropathy, the extent of tubulointerstitial fibrosis is the leading cause of end-stage renal disease; fibrosis is closely correlated with renal dysfunction. Although a wide array of medicinal plants play a role in the prevention and treatment of diabetes, there are few reports of the application of herbal medicines in amelioration of renal fibrosis, or the underlying mechanisms by which such benefits are mediated. The efficacy of the Ayurvedic antidiabetic medicine *Salacia oblonga* (SO) root on rat renal fibrosis was investigated. An aqueous extract from SO (100 mg/kg, p.o., 6 weeks) diminished renal glomerulosclerosis and interstitial fibrosis in Zucker diabetic fatty (ZDF) rats, as revealed by van Giesen-staining. SO also reduced renal salt-soluble, acid-soluble and salt-insoluble collagen contents. These changes were accompanied by normalization of hypoalbuminemia and BUN. Gene profiling revealed that the increase in transcripts encoding the glomerulosclerotic mediators collagen I, collagen IV, fibronectin, angiotensin II type 1 receptor (AT1), transforming growth factor (TGF)-**β**1, plasminogen activator inhibitor (PAI)-1 observed in ZDF rat kidney was suppressed by SO. In rat-derived mesangial cells, similar to the effect of the AT1 antagonist telmisartan, SO and its major component mangiferin suppressed the stimulatory effect of angiotensin II on proliferation and increased mRNA expression and/or activities of collagen I, collagen IV, fibronectin, AT1, TGF-**β**1 and PAI-1. Considered together the present findings demonstrate that SO attenuates diabetic renal fibrosis, at least in part by suppressing anigiotensin II/AT1 signaling. Further, it now emerges that mangiferin is an effective antifibrogenic agent.

## 1. Introduction

Fibroproliferative diseases, including cardiovascular disease, progressive kidney disease and macular degeneration, are leading causes of morbidity and mortality and can affect all tissues and organ systems. Nevertheless, despite the enormous impact of these conditions on human health, there are currently no approved treatments that directly target the mechanism(s) of fibrosis [1]. Diabetic nephropathy is the leading cause of end-stage renal disease worldwide and is an independent risk factor for all-cause and cardiovascular mortalities in diabetic patients. Diabetic nephropathy is structurally characterized by an early thickening of tubular and glomerular basement membranes due to the excessive accumulation of the extracellular matrix [2]. Excessive deposition of extracellular matrix in the glomerular mesangium and tubulointerstitium is closely associated with a progressive decline in renal function in diabetes [2, 3]. In human diabetic nephropathy, the extent of glomerulosclerosis and tubulointerstitial fibrosis are well correlated with renal dysfunction [4–6]. Accordingly, prevention and treatment of renal fibrosis may improve renal dysfunction.

It is well known that activation of the renin-angiotensin-aldosterone system is critical for development of cardiovascular and renal fibrosis in hypertension and diabetes. Although all major components of the renin-angiotensin-aldosterone system can exhibit profibrotic activity, angiotensin II, the principal constituent peptide of the renin-angiotensin-aldosterone system, plays an important role in the development of renal fibrosis [7]. Chronic angiotensin II infusion induces extensive renal fibrosis in rats [8]. The angiotensin converting enzyme inhibitor enalapril inhibits glomerular gene expression of extracellular matrix proteins in diabetic rats [9]. In mesangial cell culture, angiotensin II increases the synthesis of matrix proteins such as collagen type I and fibronectin; this can be inhibited by the angiotensin II type 1 receptor (AT1) antagonist losartan [10]. It has been suggested recently that targeting the renin-angiotensin-aldosterone system might be an effective strategy to decrease fibrogenesis in progressive renal disease [11].

Recently, there has been increased global interest in traditional medicines, including Ayurveda (traditional Indian) and Chinese medicines [12–14]. Although these medicines have been used for prevention and treatment of various disorders for several thousand years, more extensive scientific evaluation of their mechanisms of action and provision of a strong evidence base are still required. *Salacia* species (e.g., *S. oblonga, S. prinoides*, *S. reticulata*), known as “Ponkoranti” in Ayurvedic medicine, are widely distributed in Sri Lanka, India, China, Vietnam, Malaysia, Indonesia and other countries. The roots and stems of these plants have been used in the treatment of diabetes and obesity in the Ayurvedic system of Indian traditional medicine [15, 16]. Mangiferin, one of the main components in *Salacia* species, is also present in relatively abundant amounts in some fruits (e.g., mango) and other traditional anti-diabetic herbs and teas (e.g., *Anemarrhena asphodeloides*, *Mangifera indica*, *Aspalathus linearis* and *Cyclopia intermedia*) [16, 17]. Recently, *Salacia* species have been extensively consumed in Japan, USA and other countries as food supplements for the prevention of obesity and diabetes and they have been the subject of broad studies for diabetes management. It has been demonstrated that aqueous extracts of the root of *S. oblonga* Wall. (Celastraceae) (SO) activate the peroxisome proliferator-activated receptor (PPAR)-*α* [18], and ameliorate postprandial hyperglycemia, hyperlipidemia, hepatic steatosis, cardiac lipid accumulation and fibrosis in Zucker diabetic fatty (ZDF) rats [18–21]. It has not yet been evaluated whether this traditional antidiabetic herbal medicine is also beneficial in renal complications associated with diabetes.

The aims of the present study were to examine the effects and underlying mechanism of SO and its major component mangiferin on rat renal fibrosis using ZDF rats and rat-derived primary mesangial cells.

## 2. Methods

### 2.1. Chemicals and Reagents

Kits for determination of collagen (Biocolor, Brisbane, Australia), albumin (BioAssay Systems, Sydney, Australia), BUN (Sigma, Sydney, Australia) and glucose (Wako, Osaka, Japan), and uric acid (Wako, Osaka, Japan) and mangiferin (MA, Sigma, Sydney, Australia) were purchased commercially.

### 2.2. Animals and Diet

Male Zucker lean (ZL) and ZDF rats aged 13–15 weeks were obtained from Monash University Animal Services (Clayton, Victoria, Australia). Male Sprague-Dawley rats weighing 130–150 g were purchased from the Experimental Animal Center, Sun Yat-Sen University (Guangzhou, China). The animals were housed in an air-conditioned room at *23*  ±  *1*°C with a 12-h light/dark cycle and were provided *ad libitum* with water and standard pelleted diets. Animals were allowed free access to the food and water. All animal experimental procedures were in accordance with guidelines set by the National Health and Medical Research Council of Australia, and approved by the animal ethics committees of the University of Sydney, Australia or Sun Yat-Sen University, China.

### 2.3. Preparation and Identification of Aqueous SO Extract

SO roots were collected from Tamil Nadu, India and their identity was confirmed using botanical and pharmacognostic criteria. The voucher sample was deposited with the Pharmafood Institute (Kyoto, Japan; voucher No: PS0075). The aqueous SO extract was prepared as previously described [19]. The yield of the extract from the dried root was 6.5%. SO extract was characterized by HPLC [21] and the content of MA, a prominent component considered suitable for the quality assurance of *Salacia* species and its products [22], was found to be 1.4%, which is within the previously reported range (Japanese patent P2002-267655).

### 2.4. Treatment Protocol

Plasma glucose levels in non-fasted animals were determined prior to any treatment. Animals were then subdivided into experimental groups on the basis of plasma glucose level and body weight: ZL control, ZL SO, ZDF control and ZDF SO groups (five animals per group). There were no differences in plasma glucose concentrations or body weight between the two ZL groups or between the two ZDF groups; these parameters were substantially lower in ZL groups than in ZDF groups (data not shown), which is consistent with the previous reports [18–21]. SO at a dose of 100 mg/kg, which was obtained from the previous experiments [18–21], was suspended in 5% Gum Arabic and administered to animals by oral gavage once daily for 6 weeks. The same volume of vehicle (5% Gum Arabic) was administered to animals in the control group. The animals were weighed once after 3-4 days in order to determine the volumes of the test sample to be administered.

### 2.5. Measurement of Blood Biochemical Parameters and Kidney Weight

Non-fasting blood samples (anti-coagulated with heparin) were obtained from the tail veins of rats at the age of 20–22 weeks using light halothane anesthesia. Plasma samples were stored at −80°C for subsequent determination of albumin, BUN and uric acid (using the commercially available kits, listed above). The animals were euthanized by rapid dislocation of the neck vertebra. The kidneys were rapidly excised and accurately weighed. A segment of the right kidney was cut into slices, frozen in liquid nitrogen and stored at −80°C for collagen assay and gene expression analysis.

### 2.6. Renal Histology and Determination of Renal Fibrosis

The remaining kidney segment was immediately fixed in 10% neutral formalin and prepared for pathological examination. Renal fibrosis was measured essentially as described previously [19, 23]. To determine the degree of collagen fiber accumulation, forty fields in three individual sections were randomly selected and the areas of van Giesen-stained interstitial collagen deposit and the total kidney area were determined by image analysis (KS 400 Imaging System; Carl Zeiss Vision, Eching, Germany). The ratio of the areas of interstitial collagen deposit to the total kidney area was calculated. The glomerular cross-sectional areas in hematoxylin-eosin (HE)-stained slices were determined morphometrically. The cross-sectional area was measured in glomeruli that were cut transversely. The outer borders of the glomeruli were traced at 200× magnification, and the glomerular areas were calculated. Fifty glomeruli per kidney were counted, and the averaged value was used for analysis.

### 2.7. Renal Collagen Assay

The composition of collagen in renal tissue was analyzed using the commercially available Sircol Collagen Assay (Biocolor Ltd, Northern Ireland), a colorimetric method in which Sirius red dye binds to the side chains of amino acids found in collagen. The salt soluble collagen and covalently cross-linked insoluble collagen fractions were obtained by the successive treatment of rat kidney with salt soluble collagen buffer (0.05 M Tris buffer, pH 7.5, 1.0 M NaCl), acetic acid (0.5 M, pH 3.0) and Milli-Q water (with heating to 80°C, 1 h). Aliquots of extract (50 *μ*l) were mixed gently with 1 ml of Sircol dye reagent, incubated for 30 min at room temperature, and then centrifuged at 10 000 g to isolate the collagen-bound dye in precipitated material. One milliliter of alkali reagent was then added to release the collagen-bound dye into the supernatant. After vortex-mixing (5 min) and centrifugation at 10 000 g (15 min), 200 *μ*l of supernatant was transferred to 96-well plate, and the absorbance at 550 nm was measured in a micro-plate reader. The concentration was determined from standard curves, and the collagen content in the kidney tissue was expressed relative to the total dry weight.

### 2.8. Cell Culture

Primary cultures of rat mesangial cell were prepared as previously described [24–26]. Briefly, rats were euthanased by prompt dislocation of the neck vertebra. In order to prevent contamination with interstitial fibroblasts the bilateral kidney cortical tissues distant from the medulla were harvested. The tissues were minced in Hanks' balanced salt solution. The suspension was filtered using 100, 200 and 250 mesh sieves and centrifuged twice at 400 g for 2 min to obtain purified renal glomeruli. The resultant cell pellets were dispersed and incubated in HBSS containing 375 U/ml collagenase type 1 (Gibco, China) at 37°C for 15 min. After recentrifugation the cell pellet was re-suspended in a mixture of 80% RPMI 1640 medium (Gibco) and 20% fetal bovine serum containing 100 ng/ml insulin, 55 ng/ml transferrin, 50 pg/ml selenium acid and antibiotics (Gibco). The cells were incubated in an environment of 5% CO_2_–95% O_2_ at 37°C. Mesangial cells were identified by morphological characterization and immunocytochemistry; they stained positive for vimentin and *α*-smooth muscle actin and negative for factor VIII and cytokeratin expression. Cells at passage 1–4 were used in all experiments.

### 2.9. Cell Viability and Proliferation

Cell viability was determined by MTT assay. In brief, cells (1 × 10^4^ cells) were grown to 70% confluence in 48 cell culture wells and were then serum-starved for *∼*16–18 h. The medium was replaced with fresh medium containing various concentrations of SO (10, 20 and 40 *μ*g/ml), MA (25 *μ*M, Sigma, Guangzhou, China) or telmisartan (10 *μ*M, Sigma) 1  h before treatment with angiotensin II (10^−6^ M). After 20 h, MTT stock solution (5 mg/ml; Sigma) was added and 4 h later the media was removed and cells were kept in 200 *μ*l of DMSO for 15 min. The optical density at 570 nm was measured in a Gemini Microplate reader.

DNA synthesis was assayed by ^3^H-thymidine incorporation. Confluent mesangial cells in 24-well culture dishes were serum-deprived for 16–18 h. SO (10, 20 and 40 *μ*g/ml), MA (25 *μ*M) or telmisartan (10 *μ*M) was added to the medium with 10% FBS and 1 *μ*Ci/ml ^3^H-thymidine (Beijing Atom High-tech Co. Ltd, Beijing, China) 1 h before treatment with angiotensin II (10^−6^ M) and 24 h later, the cells were washed three times with cold PBS (Boster, AR0030, Wuhan, China). DNA was precipitated at 4°C by treatment with 5% trichloroacetic acid for 1 h and the pellet was dissolved in 0.1 M NaOH and absolute ethanol; radioactivity was determined by liquid scintillation counting (Beckman Counter, LS6000, Minnesota, USA).

### 2.10. ELISA Analysis of Fibronectin and Transforming Growth Factor-*β* Protein Levels in Rat Mesangial Cells

Confluent rat primary mesangial cells in 6-well culture dishes were serum-deprived for *∼*16–18 h. SO (10, 20 and 40 *μ*g/ml), MA (25 *μ*M) or telmisartan (10 *μ*M) were added to the medium in 10% FBS 1 h prior to treatment with angiotensin II (10^−6^ M). Twenty-four hours later, the medium was collected for determination of fibronectin and transforming growth factor (TGF-*β*) protein levels by ELISA (Boster) according to the manufacturer's instructions.

### 2.11. Gene Expression Analysis in Rat Kidney and Rat-Derived Mesangial Cells

Total RNA was prepared from the kidneys of individual rats or rat mesangial cells using TRIzol reagent (Invitrogen, Australia and Invitrogen, China). The relative levels of specific mRNAs were determined by reverse transcriptase polymerase chain reaction (RT-PCR), as described previously [18, 20, 21]. Single-stranded cDNA was synthesized from 1 *μ*g of total RNA using SuperScript II RNase H Reverse Transcriptase, as per the manufacturer's instructions (Invitrogen, Australia and Invitrogen, China). PCR was performed on a thermocycler (PTC-200 DNA engine, MJ Research Inc, MA, USA). The genes examined were collagen I, collagen IV, fibronectin, plasminogen activator inhibitor (PAI)-1, urokinase-type plasminogen activator (uPA), TGF-*β*1 and AT1. The sequences of the sense and antisense primers used for amplification are shown in Table 1. PCR samples were electrophoresed on 3% agarose gels and stained with ethidium bromide. The gel images were digitally captured with a CCD camera and analyzed using ImageJ 1.29x software (NIH, USA). RT-PCR values are presented as ratios of the specified gene signal in the selected linear amplification cycle relative to the signal for *β*-actin (housekeeping gene).

### 2.12. Data Analysis

All results are expressed as means ± SEM. Data were analyzed by single-factor analysis of variance (ANOVA). If a statistically significant effect was found, the Newman-Keuls test was performed to isolate the difference between the groups. *P*-values *<* .05 were considered to indicate significance.

## 3. Results

### 3.1. Renal Histology and Collagen Accumulation in ZL and ZDF Rats

**Figure 1:**
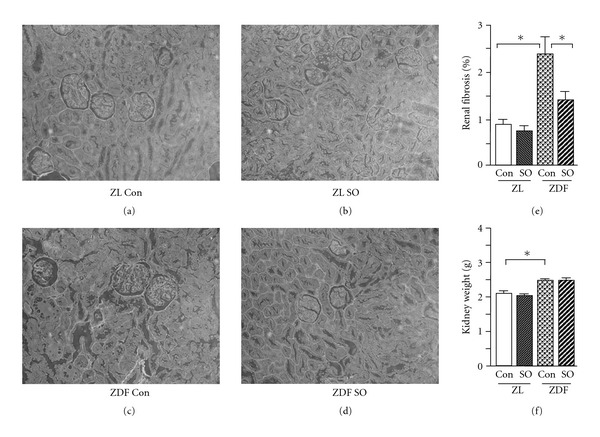
Renal fibrosis and kidney weights 6 weeks after treatment of male ZL and ZDF rats with vehicle (con) or SO extract. The renal van Giesen-stained collagen deposit area and the total renal tissue area were determined by image analysis. (a)–(d) Representative samples of renal fibrosis (red) (magnification 200×); (e) the ratio of the area of collagen accumulation to the total renal tissue area; (f) kidney weight. All values are means ± SEM (*n* = 5, each group). *P < .05.

SO has been demonstrated previously to ameliorate postprandial hyperglycemia, hyperlipidemia, hepatic steatosis, cardiac lipid accumulation and fibrosis in ZDF rats [18–21]. In the present study, the extent of renal fibrosis in ZL and ZDF rats was first assessed using the van Giesen-staining method. Renal glomerulosclerosis and interstitial fibrosis with tubular atrophy were evident in sections from ZDF rats (Figure 1(c)). The stained area (red color) was more extensive in the glomerular mesangium and tubulointerstitium of ZDF rats (Figure 1(c)), compared with ZL rats (Figure 1(a)). Thus, the area of collagen deposition as a proportion of total kidney tissue was increased in ZDF rats to 2-fold of ZL controls (*P* < .05; Figure 1(e)). The kidney weights of ZDF rats were increased by 15% over those in ZL rats (*P* < .05; Figure 1(f)). SO treatment over a 6-week period significantly diminished the extent of van Gieson-staining by 40% in ZDF rats (*P* < .05), but had minimal effect in ZL rats (Figures1(b), 1(d) and 1(e)). This treatment did not affect kidney weight in rats of either genotype (Figure 1(f)).

**Figure 2:**
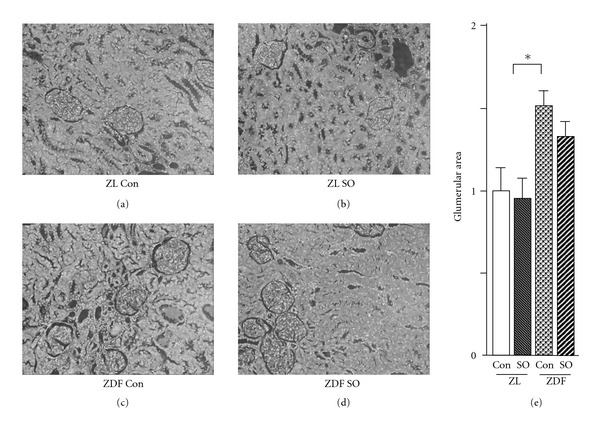
Renal tissue morphometry and glomerular cross-sectional areas 6 weeks after treatment with vehicle or SO extract in male ZL and ZDF rats. The cross-sectional area was measured in glomeruli (hematoxylin-eosin staining). (a)–(d) Representative samples (magnification 200×); (e) glomerular cross-sectional areas. All values are means ± SEM (*n* = 5, each group). *P < .05.

Glomerular cross-sectional areas in ZDF rats were 1.5-fold of those in ZL rats (*P* < .05; Figures 2(a), 2(c) and 2(e)). However, SO treatment minimally affected the glomerular cross-sectional area in kidney of ZL and ZDF rats (Figures 2(b), 2(d) and 2(e)).

**Figure 3:**
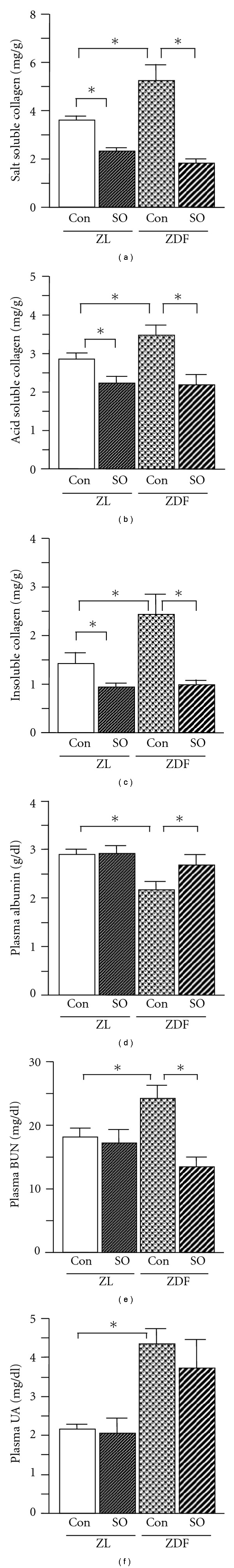
Composition of renal collagens and serum biochemical parameters reflecting renal function in rats. The composition of collagen in kidney tissue was analyzed using the colorimetric Sircol Assay. The collagen content in the kidney tissue was standardized to the total dry weight of renal tissue. The biochemical parameters were quantified enzymatically. Renal salt-soluble collagen (a), acid-soluble collagen (b), insoluble collagen (c), plasma albumin (d), BUN, (e) and plasma uric acid (f). All values are means ± SEM (*n* = 5, each group). *P < .05.

Furthermore, the effects of SO treatment on the nature of the collagens present in rat kidneys were analyzed. It was found that the quantities of total salt soluble (Figure 3(a)), acid soluble (Figure 3(b)) and insoluble (Figure 3(c)) collagens were elevated by 46, 22 and 72%, respectively, in the kidneys of ZDF rats compared with ZL control (*P* < .05). Although treatment with SO significantly decreased renal total collagen contents in all animals, the effects were more pronounced in ZDF rats than in ZL rats.

### 3.2. Biochemical Parameters Relating to Renal Function in ZL and ZDF Rats

Glomerular and interstitial injury and fibrosis may induce renal dysfunction. Consistent with previous reports [27, 28], ZDF rats were found in the present study to exhibit elevated BUN (34% increase compared with ZL control; *P* < .05) and plasma uric acid levels (to 2-fold of ZL control; *P* < .05) as well as decreased serum albumin concentrations (to 75% of ZL control; *P* < .05) (Figures 3(d)–3(f)), which is due to prolonged urinary protein excretion. Importantly, SO treatment restored albumin concentrations to those found in ZL rat plasma and also normalized BUN in ZDF rats (Figures 3(d) and 3(e)). However, SO treatment did not affect plasma uric acid (UA) concentrations in ZDF rats (Figure 3(f)), or the corresponding parameters in ZL rats.

### 3.3. Cell Proliferation in Rat-Derived Mesangial Cells

**Figure 4:**
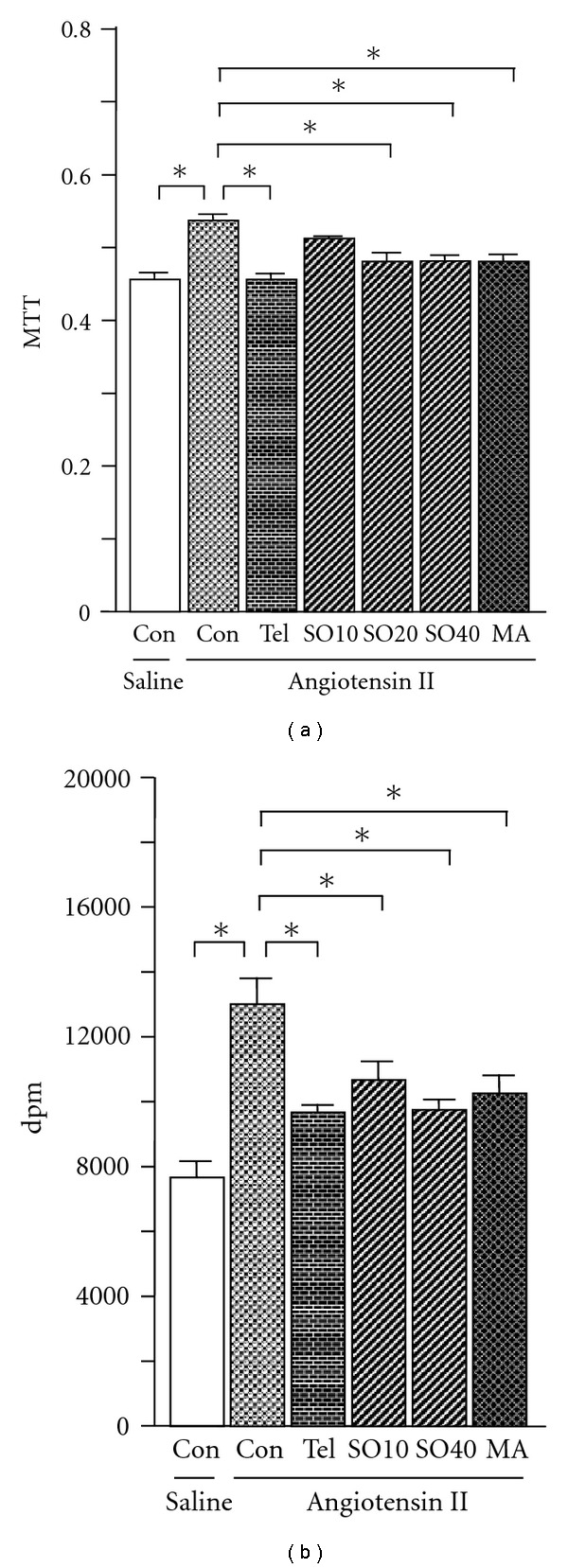
Angiotensin II-induced cell proliferation in rat-derived mesangial cells. SO (SO10: 10; SO20: 20 and SO40: 40 *μ*g/ml), MA (25 *μ*M) or telmisartan (Tel, 10 *μ*M) was added 1 h before mesangial cells were treated with angiotensin II (10^−6^ M). Cell viability was determined by MTT assay ((a), *n* = 6, each group) and DNA synthesis was assessed by ^3^H-thymidine incorporation ((b), *n* = 6, each group), as described in “Methods” section. All values are means ± SEM. *P < .05.

The effect of SO, MA and the AT1 antagonist telmisartan on angiotensin II-induced mesangial cell proliferation was assessed. Cell viability, as reflected by MTT assay, and proliferation, reflected by ^3^H-thymidine incorporation, were significantly increased (by 18 and 70%, resp.) after stimulation with angiotensin II (Figures 4(a) and 4(b)). Treatment with SO at concentrations of 20 and 40 *μ*g/ml, as well as MA (25 *μ*M) and telmisartan (10 *μ*M), all suppressed the angiotensin II-induced increase in cell viability (between 10 and 15%; all *P* < .05) and proliferation (by 18–26%; all *P* < .05), whereas the low concentration of SO (10 *μ*g/ml) showed minimal effect.

### 3.4. Renal Gene Expression Profiles

#### 3.4.1. Genes Implicated in Fibrogenesis

**Figure 5:**
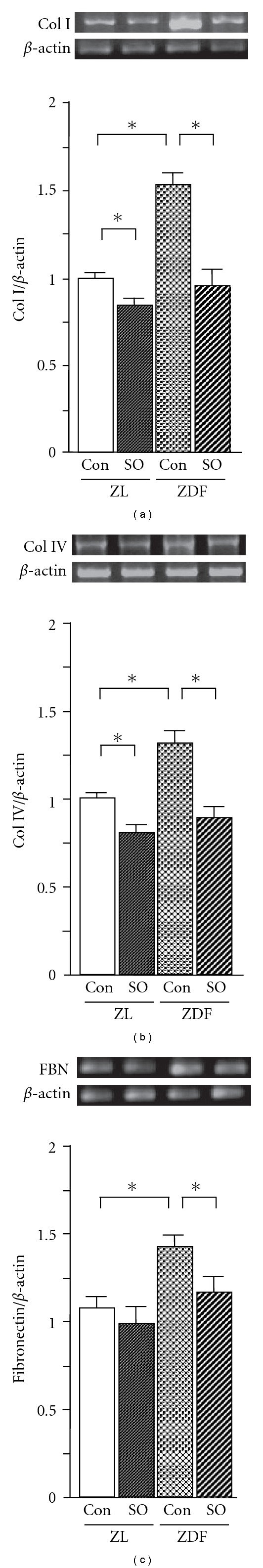
Renal expression of collagen (Col) I (a), Col IV (b), fibronectin (c) mRNAs in ZL and ZDF rats. Total RNA was extracted from renal tissues using TRIzol. The relative levels of specific mRNAs were determined by RT-PCR. Results were normalized to *β*-actin. All values are means ± SEM (*n* = 5, each group). *P < .05.

**Figure 6:**
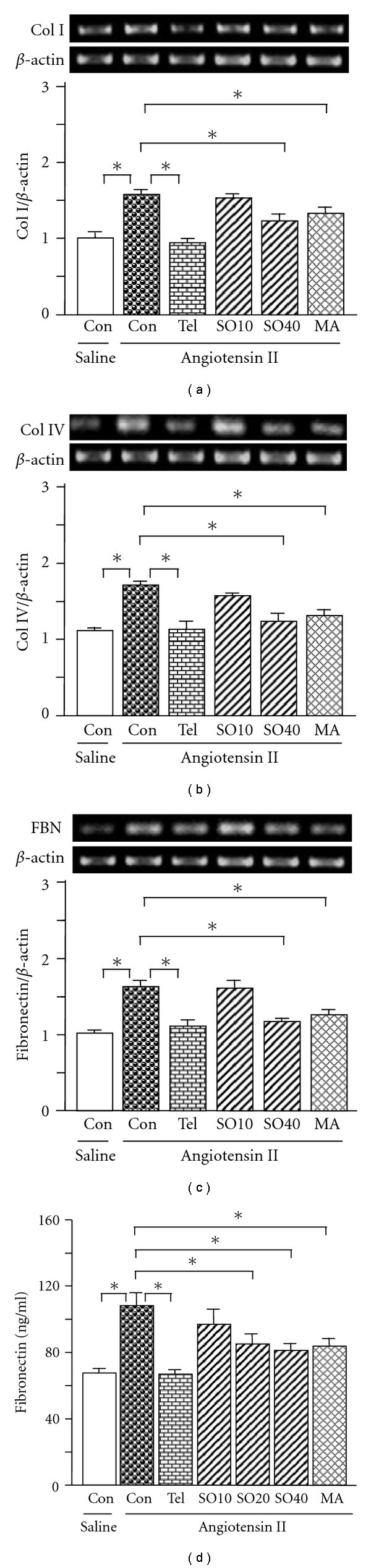
Expression of collagen (Col) I (a), Col IV (b), fibronectin (c) mRNAs and fibronectin protein (d) in rat mesangial cells. SO (SO10: 10; SO20: 20 and SO40: 40 *μ*g/ml), MA (25 *μ*M) or telmisartan (Tel. 10 *μ*M) was added 1 h before mesangial cells were treated with angiotensin II (10^−6^ M). Twenty-four hours later total RNA was extracted from the mesangial cells using TRIzol. The relative levels of specific mRNAs were determined by RT-PCR. Results were normalized to *β*-actin. In a parallel experiment, the medium was collected after mesangial cells were treated with the test agents in combination with angiotensin II. fibronectin protein was determined by commercial ELISA kit according to the manufacturer's instructions. All values are means ± SEM (*n* = 3, each group). *P < .05.

Deposition of extracellular matrix proteins including collagen I, collagen IV and fibronectin is an important component of the scarring observed during the evolution of glomerulosclerosis and tubulointerstitial fibrosis [29]. In the present study, renal expression of these fibrogenic genes was determined. Consistent with increased renal fibrosis, ZDF rats showed increased renal expression of collagen I, collagen IV and fibronectin at the mRNA level (by 55, 32 and 32%, resp.) compared with ZL-control (*P* < .05; Figures 5(a)–5(c)). SO treatment normalized the expression of all three genes when administered to ZDF rats; interestingly, collagen I and IV mRNAs were also decreased in ZL rats after SO treatment. In cultured rat mesangial cells, SO (40 *μ*g/ml), MA (25 *μ*M) and telmisartan (10 *μ*M) all effectively suppressed the angiotensin II-stimulated induction of collagen I (by 16–40%; all *P* < .05) and collagen IV (by 24–34%; *P* < .05) expression (Figures 6(a) and 6(b)). Similarly, fibronectin expression at the mRNA and protein levels were also normalized (Figures 6(c) and 6(d)).

#### 3.4.2. AT1 and TGF-*β*1 Genes

**Figure 7:**
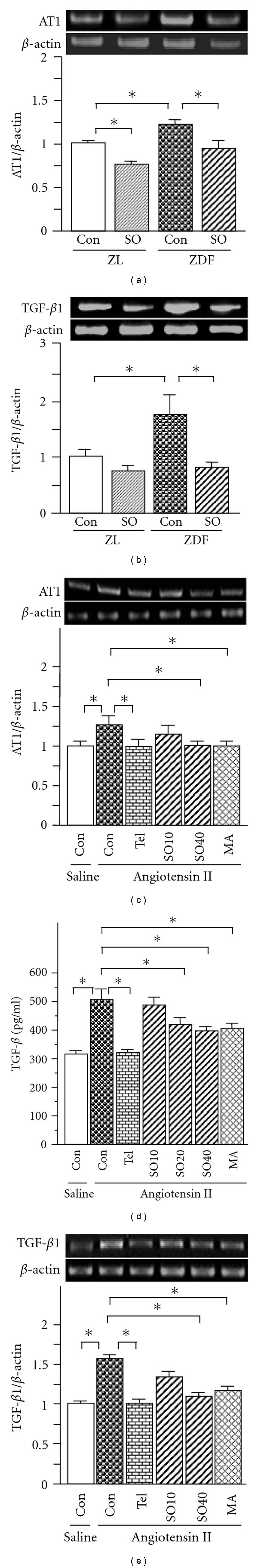
Renal expression of AT1 (a) and TGF-*β*1 (b) in ZL and ZDF rats, and levels of AT1 (c) and TGF-*β*1 (d) mRNAs, and TGF-*β* protein (e) in rat mesangial cells. In cell culture, SO (SO10: 10; SO20: 20 and SO40: 40 *μ*g/ml), MA (25 *μ*M) or telmisartan (Tel. 10 *μ*M) was added 1 h prior to treatment with angiotensin II (10^−6^ M), followed by twenty-four hours incubation. Total RNA was extracted from renal tissues and the mesangial cells using TRIzol, respectively. The relative levels of specific mRNAs were determined by RT–PCR. Results were normalized to *β*-actin. In a parallel experiment, the medium was collected after mesangial cells were treated with the test agents in combination with angiotensin II. TGF-*β* protein level was determined by commercial ELISA kit according to the manufacturer's instructions. All values are means ± SEM (*n* = 5, each group *in vivo*; *n* = 3, each group *in vitro*). *P < .05.

TGF-*β* is a key factor in glomerulosclerosis and interstitial fibrosis. The synthesis and secretion of TGF-*β*1 are activated by angiotensin II signaling through the AT1 [1] and leads in turn to enhance TGF-*β*1 signaling. AT1 and TGF-*β*1 mRNAs were both upregulated by 22 and 72%, respectively, in kidneys of ZDF rats; these increases were attenuated by SO treatment to levels comparable with those in ZL control rats (Figures 7(a) and 7(b)). In accord with these *in vivo* findings, angiotensin II treatment of mesangial cells also significantly upregulated AT1 mRNA expression by 27% (Figure 7(c)) and TGF-*β*1 mRNA and protein expression by 55 and 62% over control, respectively (Figures 7(d) and 7(e)). Treatment of cells with SO (40 *μ*g/ml), MA (25 *μ*M) and telmisartan (10 *μ*M) reversed these effects on AT1 and TGF-*β*1 and protein level in mesangial cells (Figures 7(c)–7(e)).

#### 3.4.3. Genes Implicated in Fibrinolysis

**Figure 8:**
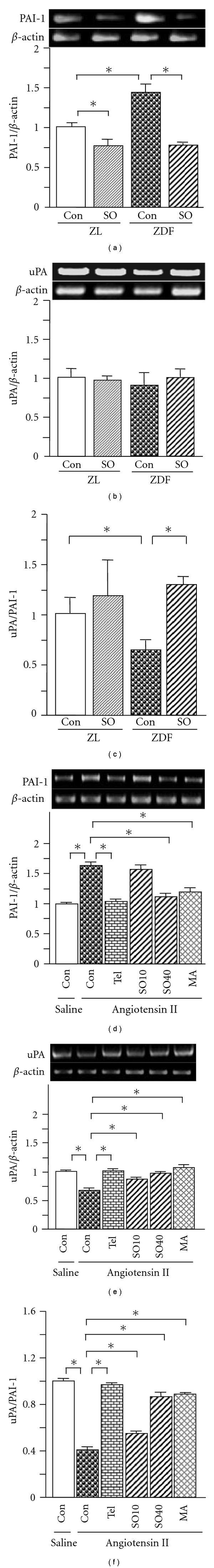
Renal expression of PAI-1 (a), uPA (b) mRNAs, and the ratio of uPA to PAI-1 (c) in ZL and ZDF rats, and levels of PAI-1 (d) and uPA (e) mRNAs, and the ratio of uPA to PAI-1 (f) in rat mesangial cells. In cell culture, SO (SO10: 10 and SO40: 40 *μ*g/ml), MA (25 *μ*M) or Tel (10 *μ*M) was added 1 h prior to treatment with angiotensin II (10^−6^ M), followed by 24-h incubation. Total RNA was extracted from renal tissues and the mesangial cells using TRIzol, respectively. The relative levels of specific mRNAs were determined by RT–PCR. Results were normalized to *β*-actin. All values are means ± SEM (*n* = 5, each group *in vivo*; *n* = 3, each group *in vitro*). *P < .05.

PAI-1 is the principal inhibitor of plasminogen activation [30] and uPA is a central activator of fibrinolysis. As part of the present study, the renal expression of these genes that participate in the synthesis and degradation of the extracellular matrix was examined. Renal PAI-1 mRNA was upregulated significantly by 43% in ZDF rats (Figure 8(a)), although renal uPA mRNA expression was unchanged (Figure 8(b)), the ratio of renal uPA to PAI-1 mRNAs was decreased by 29% (*P* < .05; Figure 8(c)). SO treatment of ZDF rats and, to a lesser extent, ZL rats significantly decreased PAI-1 mRNA by 46 and 15%, respectively, but was without effect on uPA mRNA expression. Thus, there was a corresponding increase in the uPA : PAI-1 ratio in SO-treated animals that was more pronounced in ZDF rats (88% increase) than in ZL rats (18% increase) (both *P* < .05; Figure 8(c)). Findings in mesangial cells were consistent with *in vivo* findings. Similar to telmisartan (10 *μ*M) (decreased by 36.7%, *P* < .05), SO (40 *μ*g/ml) and MA (25 *μ*M) significantly suppressed the angiotensin II-mediated increase in PAI-1 mRNA (*P* < .05; Figure 8(d)) and prevented the angiotensin II-induced decline in uPA mRNA expression (*P* < .05; Figure 8(e)) and the uPA : PAI-1 mRNA ratio (Figure 8(f)).

## 4. Discussion

The present study clearly demonstrates that SO treatment attenuates the renal interstitial fibrosis, glomerulosclerosis and collagen deposition in ZDF rats, as revealed by van Giesen-staining. Consistently, the elevated renal expression of fibrogenic genes including collagen I, collagen IV and fibronectin in ZDF rats, was restituted by SO treatment. Moreover, SO treatment ameliorated the increase in both insoluble and soluble collagens in the kidneys of ZDF rats. These improvements were accompanied by the normalization of BUN and hypoalbuminemia, findings that are consistent with the attenuation of diabetic nephropathy.

Angiotensin II is a renal growth factor, inducing hyperplasia and hypertrophy in a cell type-dependent fashion. Angiotensin II is a key factor in the inflammatory and fibrotic response in kidney diseases [31]. This vasoactive peptide activates mesangial and tubular cells and interstitial fibroblasts, increasing the expression and synthesis of extracellular matrix proteins, such as collagen I and fibronectin [7, 10, 32, 33]; these effects are mediated by angiotensin II via the AT1 [10].

Glucose has been shown to increase the production of angiotensin II, thereby decreasing collagenase activity and increasing collagen deposition; the latter effect was prevented in rat mesangial cells by the AT1 antagonist losartan [34]. In ZDF rats, AT1 mRNA and protein expression was increased along with the associated glomerulopathy, upregulation of the extracellular matrix protein fibronectin, renal inflammation and proteinuria; again, these effects were attenuated by losartan [28]. Thus, angiotensin II signaling is stimulated in this animal model and leads to renal dysfunction and fibrogenesis. The present study demonstrated that the overexpression of the AT1 gene in ZDF rat kidney was normalized by SO treatment. Furthermore, SO and its major constituent MA suppressed a range of angiotensin II-induced cellular endpoints including hyperproliferation, overexpression of collagen I, collagen IV and fibronectin mRNAs, and increased fibronectin protein level in isolated rat mesangial cells. Angiotensin II-dependent induction of AT1 gene expression was also suppressed. These effects were similar to those elicited by the AT1 antagonist telmisartan. Thus, the present findings assert that SO modulates angiotensin II/AT1 signaling, which in turn attenuates cell proliferation and fibrogenesis in ZDF rat kidney, and that MA is a candidate active constituent present in SO extracts.

TGF-*β* is a key factor in glomerulosclerosis and interstitial fibrosis that acts directly, by stimulating synthesis of extracellular matrix components and reducing collagenase production, and indirectly through other profibrogenic factors. Furthermore, TGF-*β* is important for the proliferation of intrarenal fibroblasts and the epithelial-mesenchymal transition through which tubular cells become fibroblasts [35]. Numerous studies have demonstrated that TGF-*β* plays a pivotal role in experimental diabetic kidney disease and human diabetic nephropathy [32]. Angiotensin II stimulates TGF-*β* expression in the kidney by several mechanisms and upregulates receptors for TGF-*β* [35]. Apart from its effects on TGF-*β*1 secretion and activation, angiotensin II also directly enhances TGF-*β*1 signaling [1]. Glucose-induced TGF-*β*1 secretion was suppressed by losartan in rat mesangial cells [34]. Thus, angiotensin converting enzyme inhibitors and AT1 antagonists are two classes of drugs that have the potential to inhibit angiotensin II-mediated TGF-*β* expression [35]. In the present study, SO treatment reversed the increase in renal TGF-*β*1 expression in ZDF rat kidney. Moreover, similar to telmisartan, both SO and MA inhibited the increase in TGF-*β* secretion and angiotensin II-induced TGF-*β*1 gene expression in rat mesangial cells.

TGF-*β*1 activates the plasmin system by stimulating PAI-1 gene expression [30]. PAI-1 has a number of important roles in pathological processes, including the inhibition of fibrinolysis, regulation of extracellular matrix turnover and activation of proenzymes and latent growth factors that promote tissue fibrosis and sclerosis [36]. In progressive renal diseases, such as diabetic nephropathy, PAI-1 has been identified as a critical mediator of glomerulosclerosis and interstitial fibrosis [36, 37]. The altered uPA to PAI-1 ratio reflects a change from a profibrinolytic to an antifibrinolytic state [38]. The shift toward the uPA-enriched profibrinolytic state favors renal collagen degradation. The present finding that the increase in PAI-1 gene expression in ZDF rat kidney was suppressed by SO treatment is consistent with the SO-mediated decrease in renal collagen contents. In mesangial cells, SO, MA and telmisartan treatment not only suppressed angiotensin II-induced overexpression of PAI-1, but also restituted uPA gene expression that had been suppressed by angiotensin II; thus, the mRNA ratio of uPA to PAI-1 was normalized. These findings were consistent with analogous measurements in ZDF rats.

Because glucose stimulates the production of angiotensin II [34], it is conceivable that the reported improvement of hyperglycemia by SO treatment [19] may also contribute to the attenuation of renal fibrosis in ZDF rats by decreasing renal angiotensin II. However, it is also pertinent that recent studies have provided compelling evidence that other components of the renin-angiotensin-aldosterone system, including angiotensin III, renin, and aldosterone also activate the TGF-*β* system [35]. It is now important to investigate whether other mechanisms apart from the angiotensin II/AT1 signaling pathway may also contribute to the antifibrogenic activity of SO and its active constituent MA. In this regard it is noteworthy that the SO extract also contains other active components, such as salacinol, kotalanol and kotalagenin 16-acetate [17]. The potential contributions of these constituents to the biological actions of SO could be considered in future studies.

## 5. Conclusion

**Figure 9:**
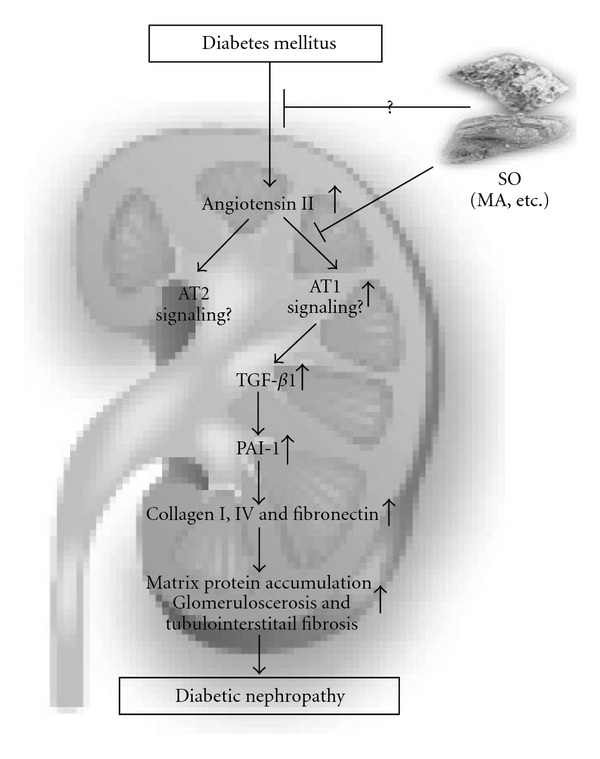
Proposed model showing SO- and MA-dependent effects on the angiotensin II/AT1 signaling pathway and fibrogenic mediators in diabetic rat kidneys.

The present findings demonstrate for the first time that a traditional antidiabetic herbal medicine attenuates renal fibrosis in ZDF rats, at least in part, by suppressing angiotensin II/AT1 signaling, possibly as proposed in Figure 9. Furthermore, it now emerges that MA is a candidate antifibrogenic agent. These studies provide new insights into the potentially important renoprotective effects and the underlying molecular mechanism of action of SO.

## Funding

Pharmafood Institute, Kyoto, Japan.

## Figures and Tables

**Table 1:** Sequences of primers used in the amplification of renal genes by RT-PCR

Gene	Accession no. or reference	Forward primer	Reverse primer	Size (bp)	Temp. (°C)
Collagen I	Li et al. [27]	5′-CATAAAGGGTCATCGTGGCTTC-3′	5′-GTGATAGGTGATGTTCTGGGAG-3′	*∼*500	62
Collagen IV	Li et al. [27]	5′-GCAGGTGTGCGGTTTGTGAAG-3′	5′-AGCTCCCCTGCCTTCAAGGTG-3′	328	55
Fibronectin	NM019143	5′-CAAGACCATACCTGCCGAAT-3′	5′-CCGTGTAAGGGTCAAAGCAT-3′	417	63
AT_1_	NM030985	5′-GAGAGGATTCGTGGCTTGAG-3′	5′-TAAGTCAGCCAAGGCGAGAT-3′	464	62
TGF-*β*1	NM021578	5′-GACCTGCTGGCAATAGCTTC-3′	5′-GACTGGCGAGCCTTAGTTTG-3′	468	58
PAI-1	M24067	5′-CCCTTCCAGAGTCCCATACA-3′	5′-CAGGCGTGTCAGCTCATTTA-3′	485	60
uPA	NM013085	5′-CAGATCCGATGCTCTTAGCC-3′	5′-GCTGCTCCACCTCAAACTTC-3′	458	61
*β*-actin	NM031144	5′-AGCCATGTACGTAGCCATCC-3′	5′-CTCTCAGCTGTGGTGGTGAA-3′	228	60

Temp, temperature.
